# Evaluating Potential Spectral Impacts of Various Artificial Lights on Melatonin Suppression, Photosynthesis, and Star Visibility

**DOI:** 10.1371/journal.pone.0067798

**Published:** 2013-07-05

**Authors:** Martin Aubé, Johanne Roby, Miroslav Kocifaj

**Affiliations:** 1 Département de physique, Cégep de Sherbrooke, Sherbrooke, Québec, Canada; 2 Département de chimie, Cégep de Sherbrooke, Sherbrooke, Québec, Canada; 3 Astronomical Institute, Slovak Academy of Sciences, Dúbravská 9, Bratislava, Slovak Republic; University of Texas Southwestern Medical Center, United States of America

## Abstract

Artificial light at night can be harmful to the environment, and interferes with fauna and flora, star visibility, and human health. To estimate the relative impact of a lighting device, its radiant power, angular photometry and detailed spectral power distribution have to be considered. In this paper we focus on the spectral power distribution. While specific spectral characteristics can be considered harmful during the night, they can be considered advantageous during the day. As an example, while blue-rich Metal Halide lamps can be problematic for human health, star visibility and vegetation photosynthesis during the night, they can be highly appropriate during the day for plant growth and light therapy. In this paper we propose three new indices to characterize lamp spectra. These indices have been designed to allow a quick estimation of the potential impact of a lamp spectrum on melatonin suppression, photosynthesis, and star visibility. We used these new indices to compare various lighting technologies objectively. We also considered the transformation of such indices according to the propagation of light into the atmosphere as a function of distance to the observer. Among other results, we found that low pressure sodium, phosphor-converted amber light emitting diodes (LED) and LED 2700 K lamps filtered with the new Ledtech’s *Equilib* filter showed a lower or equivalent potential impact on melatonin suppression and star visibility in comparison to high pressure sodium lamps. Low pressure sodium, LED 5000 K-filtered and LED 2700 K-filtered lamps had a lower impact on photosynthesis than did high pressure sodium lamps. Finally, we propose these indices as new standards for the lighting industry to be used in characterizing their lighting technologies. We hope that their use will favor the design of new environmentally and health-friendly lighting technologies.

## Introduction

The development of artificial lighting technologies over the centuries has transformed human civilization and shaped the way we live. The world has become awash with artificial lighting both during the day and at night, indoors and outdoors, from office buildings to streetlights. Before Edison’s invention of the light bulb (1879), people spent most of their time outdoors, receiving adequate daily doses of natural, full-spectrum sunlight during the day while spending their evenings and nights in relative darkness. With the growing availability of artificial lighting, people are spending an increasing amount of time inside under artificial lighting and consequently reducing the amount of time they are exposed to natural full-spectrum light during the day and darkness during the night. Around 99% of the population of the United States and Europe, and 62% of the world’s remaining population, are exposed to artificial light at night (ALAN), the amount of which is increasing rapidly each year [1]. Not only are humans but also fauna and flora are exposed to ALAN, with ensuing environmental consequences. ALAN is one of the fastest growing and most common kinds of environmental pollution. The effects of ALAN on fauna have been well defined and documented, and almost only negative effects have been reported. ALAN affects behavior, foraging, reproduction, communication, breeding cycles and the habitat of many nocturnal species [2], [3], [4], including invertebrates [5] amphibians [6], birds [7], bats [8], turtles [9], [10], [11], fish [12] and reptiles [13]. On the other hand, the impact of ALAN on flora is less documented; a review on the topic is reported by Briggs [14]. Exposure to artificial light prevents many trees from adjusting to seasonal variations. The presence of ALAN stimulates photosynthesis at a time when photosynthesis does not normally occur. Similar to humans and animals, plants require a specific cycle of light/darkness in order to grow healthily. Light affects several plant processes, such as seed germination, stem elongation, leaf expansion, conversion from a vegetative to a flowering state, flower development, fruit development, cessation of leaf production (bud dormancy) and leaf senescence and abscission; for all these processes, the duration, wavelength and intensity of the light are crucial [14]. Some of this knowledge is commonly used by the greenhouse industry to promote flowering and growth, and to stimulate fruit, vegetable and plant production. High intensity discharge (HID) lamps are popular for a large area of lighting applications in horticulture.

Artificial light is sometimes beneficial and sometimes detrimental to human health. Light has a profound impact on circadian systems and physiological functions. Because of this major impact and the growth in ALAN, there is evidence for a strong link between exposure to ALAN and disease. ALAN may be associated with an increased risk of breast cancer [15], [16], [17], [18], [19], [20], prostate and colorectal cancer [21], [22], and may also cause obesity [23], [24], diabetes [25] and depression [26]. Kloog et al. [20] report that women have 30–50% higher risk of breast cancer in the countries with the highest exposure to ALAN compared to those with the lowest exposure. One efficient way to monitor the effect of light on circadian systems is to evaluate melatonin suppression in biofluids. Melatonin, also called the sleeping hormone, is produced by the pineal gland and is released mainly during the night. Light induces a decrease in pineal melatonin hormone production and secretion; and it may also induce a phase shift in daily rhythms [27]. The discovery of a novel non-visual photoreceptor, with the photopigment melanopsin acting on our circadian function [28], [29], has changed the understanding of that mechanism. Melanopsin responds to light by decreasing pineal melatonin hormone production, with a maximum spectral sensitivity at blue wavelengths. Two light variables, intensity and wavelength [30], are responsible for the suppression of melatonin, and an illuminance of only 1.5 lux may disrupt circadian rhythms [31]. According to our measurements, taken in Sherbrooke (Canada), an illuminance of ∼2 lux is frequently encountered in urban bedrooms. Moreover, the human circadian system responds to millisecond flashes of light, delaying pineal melatonin production [32].

Indoor artificial lighting can be beneficial to humans. For example, light therapy is commonly prescribed by doctors against seasonal affective disorder [33]. It has also been shown that blue-enriched light during the day increases performance, vigilance and sleeping patterns [34]. Exposure to compact fluorescent light (CFL) at a correlated color temperature (CCT) of 6500 K (blue-enriched light) induced greater melatonin suppression, together with enhanced subjective alertness, well-being and visual comfort [35]. These results suggest that the selection of CFL with different CCT has a significant impact on circadian physiology and cognitive performance at home and at work. Finally, the availability of electronic devices with backlit screens, which are often used at night, is rapidly increasing throughout the world. In comparison with backlit liquid crystal display (LCD), evening exposure to a light emitting diode (LED)-backlit computer screen (blue-enriched light) resulted in attenuated salivary melatonin and sleepiness levels, with a concomitant increase in cognitive performance associated with sustained attention and with working and declarative memory [36]. With the progress of LED technologies, it will be important to build electronic device screens in accordance with the circadian cycle [36].

**Figure 1:**
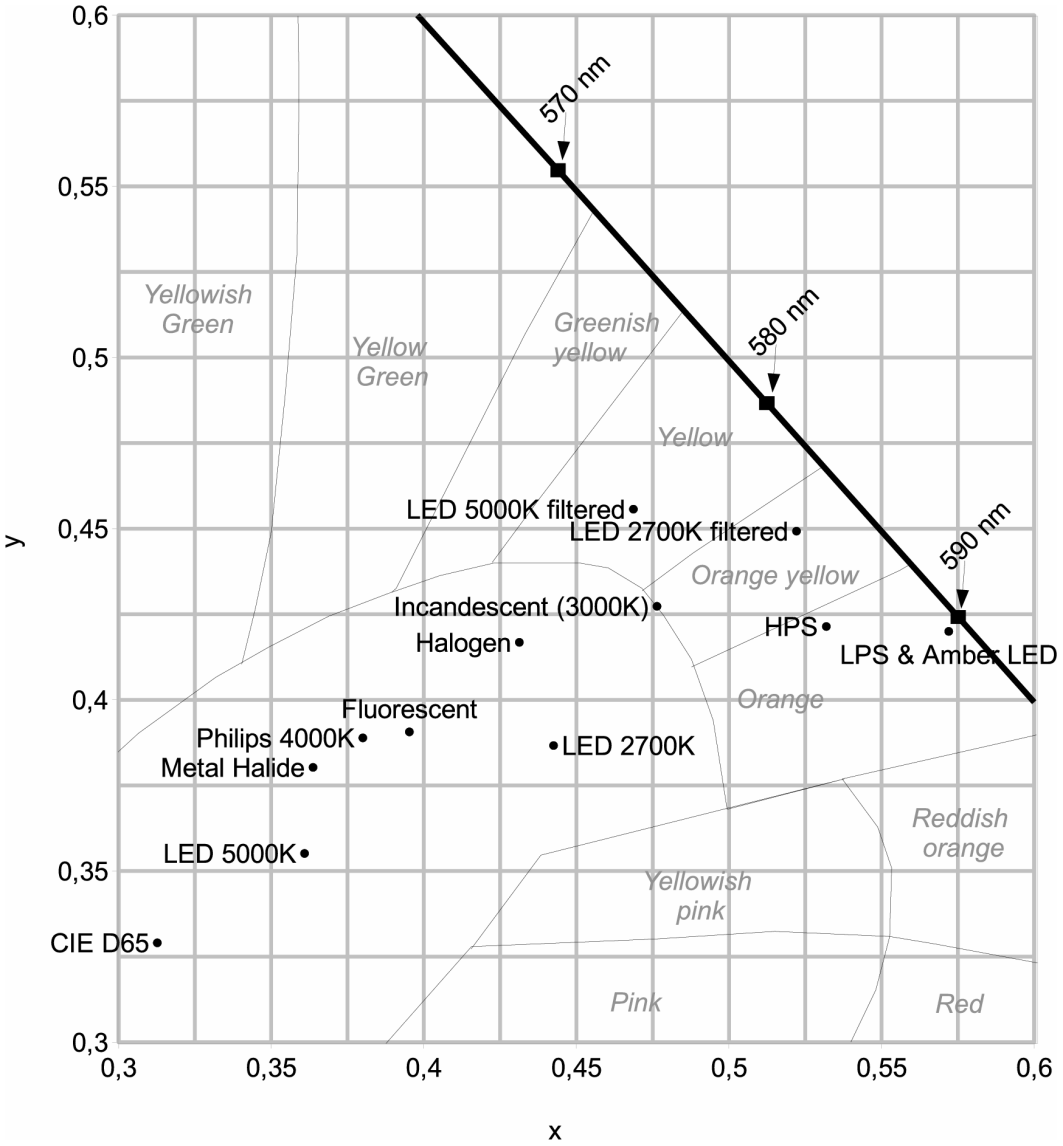
x and y chromaticity coordinates [52] for each lamp listed in Table 2. On that figure, the spectral locus, which is the line for monochromatic light, is shown by the thick black line. Thin black lines indicate color zones. Black squares show monochromatic values, while small black circles are lamps.

Since the 1960s, outdoor artificial lighting has progressively changed from incandescent-bulbs (orange-yellow color, see Fig. 1) to a high pressure sodium form (HPS, orange) and more recently to LED (blue-enriched white light). The indoor artificial lighting that is most used is cool-white fluorescent lighting (FL) for public areas and incandescent, halogen and CFL bulbs for private areas with a large span of CCT. In lighting engineering, lower CCT (CCT<5000 K) is often called warm white light while high CCT (CCT>5000 K) is called cool white light. The use of artificially generated full spectrum daylight for human activities is not common, but they are used in the field of light therapy, greenhouse lighting and for pet shops. This kind of light is reputed to mimic natural sunlight, but it is not exact in this, as will be discussed later.

Reduction in star visibility, one of the best known impacts of outdoor ALAN, has been identified by astronomers. A first abatement for the protection of night sky quality over professional astronomical observatories was adopted in 1958 in the vicinity of Flagstaff, AZ, USA. Astronomers have always preferred the use of low pressure sodium (LPS) lamps. This technology shows quasi-monochromatic spectral power distribution (SPD) in the orange part of the visible spectrum. This kind of SPD is easy to filter out using optical filters and its color is not very efficient in terms of atmospheric scattering. In fact, when light travels into the atmosphere, it is partly scattered by molecules and aerosols and can be redirected toward an observer looking at the stars. This astronomical light pollution is then competing with the faint light coming from the universe. According to the Mie and Rayleigh scattering theories, blue light is scattered more efficiently than other colors (e.g. blue scattering is about one order of magnitude more efficient than red scattering).

The first main difference between daytime and nighttime natural light is the intensity level, since sunlight, starlight and moonlight are not so different in terms of their relative SPD. Sunlight is around five to nine orders of magnitude brighter than typical ancestral nighttime illumination (natural or human-made). In modern times, nighttime artificial illumination in lit areas is typically four orders of magnitude higher than illumination from a natural starry sky without moonlight, and around one to two orders of magnitude higher in comparison to full moonlight illumination. Light from wood/oil burning, which was the most intense source of human-made lighting for centuries, contains a very low blue light contribution in comparison to sunlight. Nowadays, human-made light shows important differences in comparison to wood/oil burning and the Sun’s SPD. The most significant nighttime natural lights, such as those from the stars, moon and wood/oil burning, can be described as a quasi black body spectrum showing a predominant continuum SPD, while many modern artificial lights include the addition of discrete spectral lines with a very low continuum contribution. Natural light contains all wavelengths of the visible spectrum while some artificial lights contain only a subset or are dominated by a few spectral lines. Artificially generated full spectrum daylight lamps, which were designed to approximate to sunlight, contain essentially all the wavelengths of the visible spectrum but with relatively important discrete spectral lines superimposed to a continuum. Tungsten incandescent technology SPDs (halogen tungsten incandescent and standard tungsten incandescent) are similar to natural light in terms of the relative importance of the continuum, but with a lower CCT compared to the sun, moon and the brightest stars. In other words, tungsten-based SPD shows a higher relative red contribution compared to the sun. Even if, with passing time, humans are increasing their light spill into the environment, not enough consideration has been given to the development of lighting devices that have an SPD comparable to natural light (either daytime or nighttime).

In the field of lighting engineering, the parameters used to describe light spectra are very crude and do not characterize SPD in detail. As an example, CCT and the color rendering index (CRI) are often used, but both parameters refer to a black body style of spectrum. CCT is the black body temperature that gives the same color sensation to the average human eye. CRI gives information concerning to what extent a lamp spectrum can be compared to a black body SPD in terms of its color rendering. A CRI value of 100 means that the lamp SPD is a perfect black body spectrum. CRI can be evaluated by comparing the color rendering of the source with a black body of the same CCT. This is why tungsten halogen shows CRI∼100, which is the optimal value of CRI. As stated above, most modern artificial light SPDs are very far from black body values. To obtain a good approximation of human eye color perception under sunlight, we need a CRI of 100 and a CCT of around 5800 K. One technology that is not far from this ideal target is the ceramic metal halide lamp (CCT∼5400 and CRI∼96), if we ignore spectral lines superimposed on the continuum spectrum.

Recently, LED technology from the field of solid state physics has been introduced to the lighting industry. LED emits a quasi-monochromatic SPD with a typical full width at half maximum (FWHM) of the order of 30 nm and a nominal wavelength depending on the material used to make the diode junction. Nowadays, the most efficient LEDs are the blue ones with a nominal wavelength ranging from 440 nm to 480 nm. Such light have of course a CCT and CRI that are very far from natural solar radiation. To overcome this drawback, a phosphorous material is placed between the blue LED and the observer. The role of that phosphorous material is to expand the narrow SPD of the blue LED into a broad band SPD. The resultant light is almost white but, when observing it with a spectrometer, one can clearly see that the white LED SPD can be described as including the addition of a broadband yellowish SPD with a significant remaining narrow band blue SPD.

**Figure 2:**
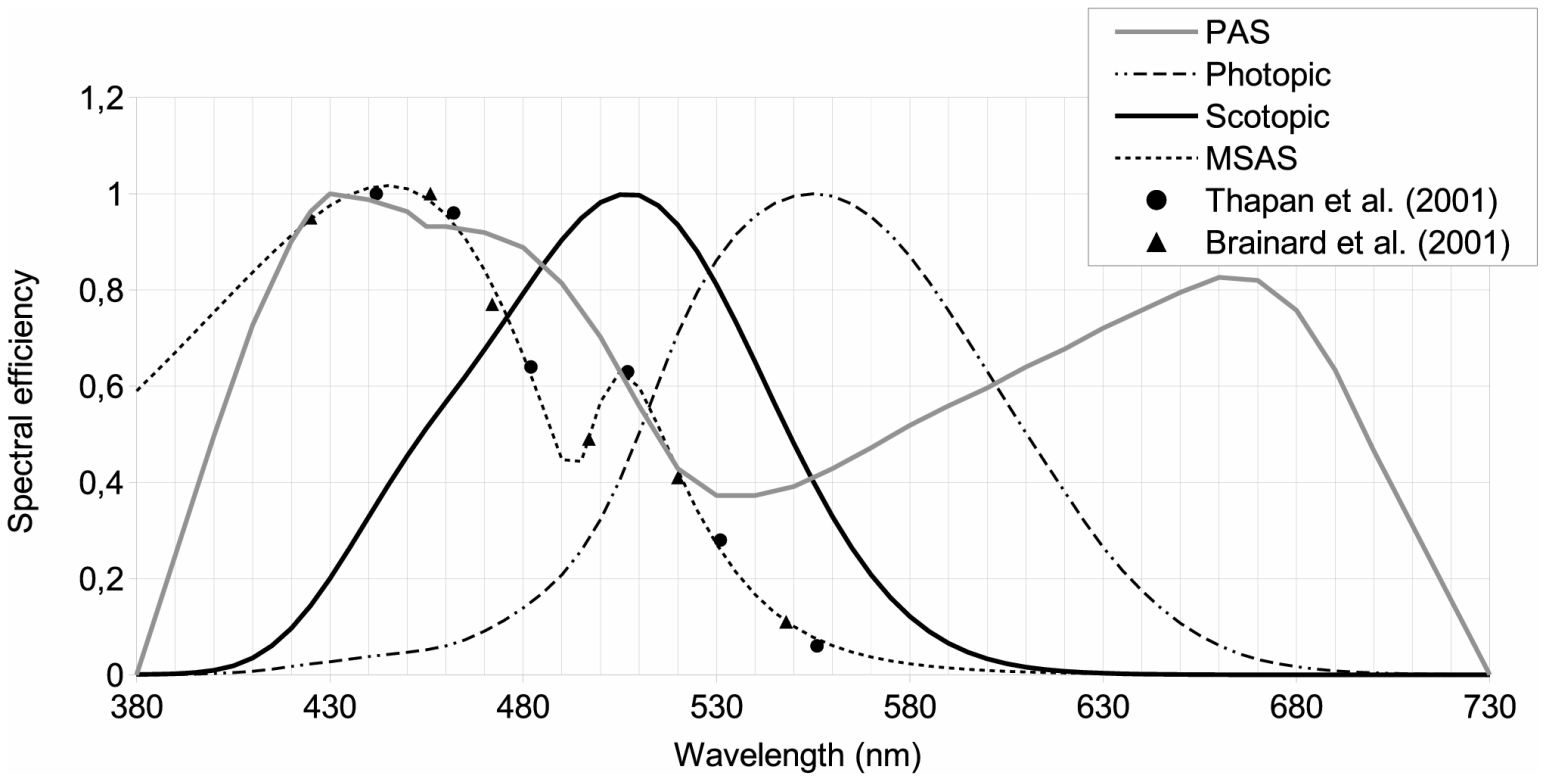
Various spectral sensitivities: human eye photopic [44] and scotopic [41] spectral sensitivity; MSAS: human melatonin suppression action spectrum; PAS: photosynthesis action spectrum.

The impact of artificial light on photosynthesis, on star visibility and on melatonin suppression is closely related to the concordance of the given spectral sensitivity of the phenomena being considered with the spectrum of the light. As an example, the photosynthesis action spectrum (PAS), or 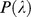, which represents the efficiency of each wavelength in inducing photosynthesis for averaged vegetable species, shows two peaks: one in the blue region at around 450 nm and the other in the red part of the spectrum at around 660 nm (see Fig. 2). Basically this means that an artificial light having a significant emission around these wavelengths is more likely to interfere with photosynthesis, especially during the night when there is no solar light. White LEDs are somewhat problematic for nighttime photosynthesis because their blue peak fits almost perfectly with the blue sensitivity peak of PAS.

The same kind of analysis can be made to estimate: 1- the impact of ALAN on star visibility by considering the low illumination eye spectral sensitivity (scotopic response), and 2- the potential impact of artificial light on circadian cycle disruption using the melatonin suppression action spectrum (MSAS).

In this paper, we will introduce three new parameters or indices to characterize a light spectrum in terms of its potential impact on respective biological processes: 1- melatonin suppression, 2- photosynthesis, and 3- scotopic vision. Our indices are intended to separate SPD from other factors acting on the given biological process. As an example, a minimum illumination is required to induce circadian cycle disruption, but our new index ignores this minimum illumination level. By using such an index, we will therefore have to assume that all other variables known to have an impact on the given biological process are favorable. In this way, the indices only deal with the potential impact of SPD shape. After defining the indices, we apply them to a variety of existing lighting technologies. We finally calculate the impact of atmospheric light scattering on indices values as a function of the distance between the light source and the observer, with and without cloud cover. All comparisons are made considering a constant lumen output for each lamp.

## Materials and Methods

### Lamp Spectral Power Distribution Data

**Figure 3:**
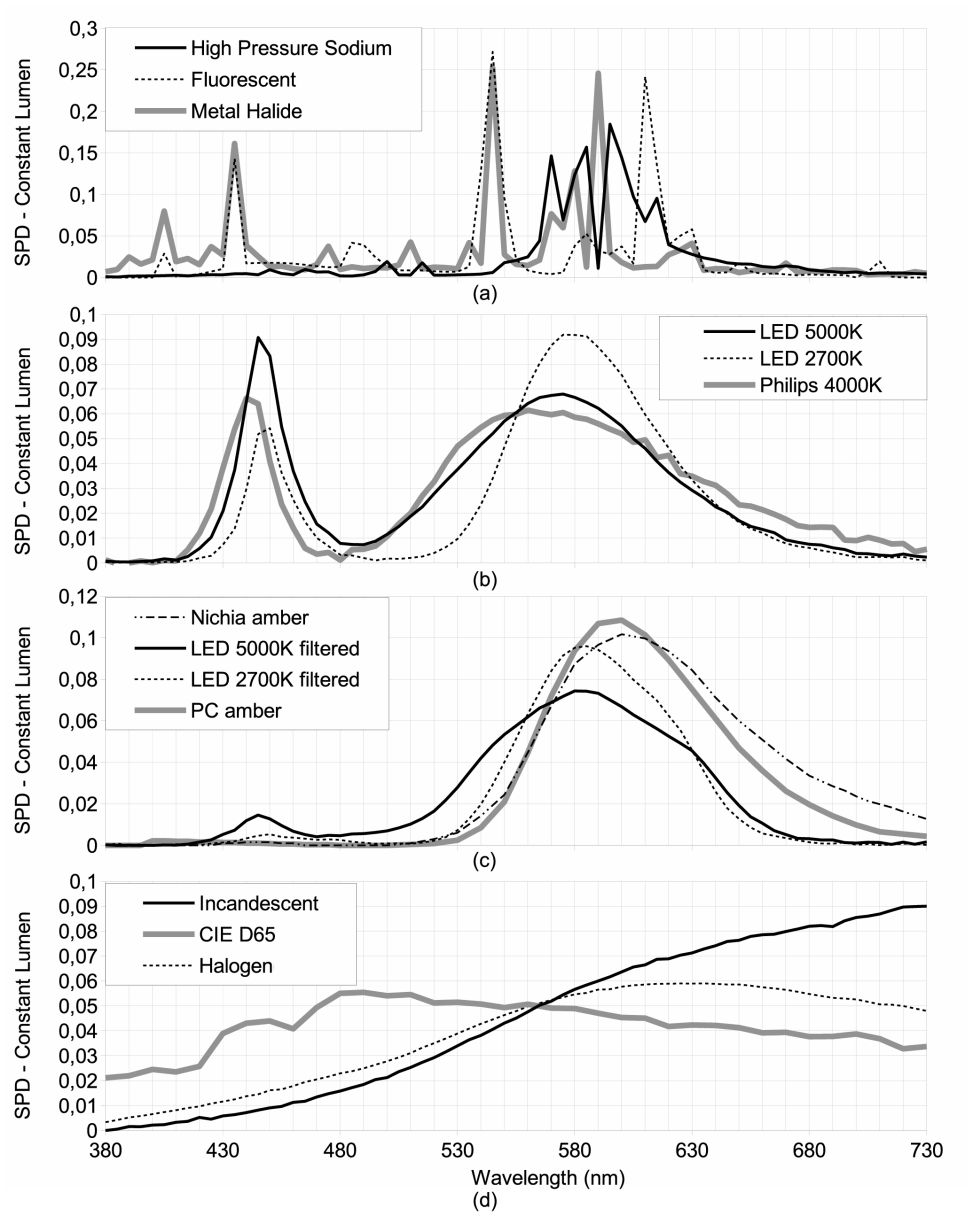
Constant lumen spectral power distributions. A subset of the spectrum used in this paper is shown here. Pane (a) shows HID lamps, pane (b) illustrates white LEDs, (c) shows low blue content broadband LEDs and (d) shows thermalized spectra such as for halogen and incandescent lamps and our reference D65 illuminant from the Commission Internationale de l’Éclairage (CIE). D65 illuminant corresponds to a midday Sun in Western/Northern Europe with CCT∼6500 K. LED filtering was performed using the *Equilib* optical filter commercialized by Ledtech International.

This experiment was conducted on the basis of a Lamp SPD Database (LSPDD) available online [37] and maintained by our research group. This dataset aims to provide independent information about the spectral characteristics of commercial lamp products. Among other information, we distribute SPDs in ASCII text format, allowing any other researchers to use this data for their own research. LSPDD is released under Creative Commons BY-NC-ND license. Some examples of SPDs from this database are shown in Figure 3.

### Spectral Sensitivities

Various spectral sensitivities have been used for this study. The first, photopic spectral sensitivity 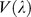, was used in order to normalize the spectrum (see section on constant lumen normalization). The three other curves, which are 1- the melatonin suppression action spectrum (MSAS), 2- the photosynthesis action spectrum (PAS) and 3- the scotopic spectral sensitivity 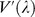, were used to calculate the spectral impact on related biological processes. In the following sections, we will detail the choice of spectral sensitivity curves with respect to each process.

#### Melatonin suppression action spectrum

In 2001, data concerning the melatonin suppression action spectrum in the spectral range from 425 nm to 560 nm were published [28], [29] (triangles and circles on Fig. 2). MSAS represents to what extent each wavelength is efficient in suppressing melatonin production. Unfortunately, the dataset provided is quite small (only 12 data points) and contains no information about MSAS values in the very deep blue and UV-A regions (no data below 425 nm). In order to generate MSAS all over our spectral range, from 380 nm to 730 nm, we tried to fit the data using a combination of two log normal curves (see Eq. 3). We chose a 2 lognormal curves to capture a breakdown in the slope from the point at 507 nm, assuming this data to be reliable. Previous authors (e.g. [29]) used a vitamin A1 retinaldehyde photo pigment template to fit the data. They obtained a correlation of R^2^ = 0.91 with 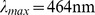. Our 2 lognormal fit is shown by the dotted curve on Figure 2. The correlation of that fit was very good with R^2^ = 0.997 and we obtained 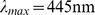. The fitted function and parameters are given in Table 1 and in Equation 1 (wavelength 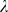 is in nm):
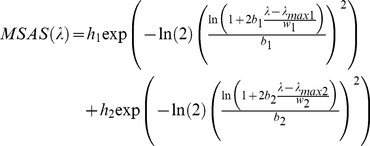
(1)where *b* represents asymmetry parameters, *w* the profile widths, 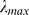 the maximum wavelengths and *h* the function heights.

**Table 1:** Parameters of the lognormal components of the MSAS fit.

Log normal #1		Log normal #2	
*h_1_*	1.017	*h_2_*	0.5239
*λ_1_*	444.7	*λ_2_*	509.3
*w_1_*	111.9	*w_2_*	30.18
*b_1_*	−0.5785	*b_2_*	0.6666

Since no constraint was exerted on the fit for wavelengths lower than 425 nm, we were unable to determine if the fitted MSAS remained good in this part of the spectrum, but we assumed that to be the case.

#### Photosynthesis action spectrum

Photosynthesis is a process in which plants, algae and some bacteria transform solar light into organic compounds. To infer the possible impact of artificial lighting on photosynthesis, we needed to define the action spectrum to be used. Many authors have shown that action spectra change from one species to another (e.g. [38]). Chlorophyll a and chlorophyll b are the two most abundant pigments in plants but we know of four other structures of chlorophyll molecules that are adapted to various environmental characteristics. As an example, chlorophyll d is found in cyanobacteria and is more sensitive to far-red light (∼710 nm). This particularity is useful, especially in a scattering medium, such as moderately deep water, where shorter wavelengths are removed by scattering along the light path. Chlorophyll a is common to all vegetable species, while chlorophyll b is mostly found in plants. These two pigments absorb more efficiently the blue and red part of the spectrum. This is in turn the reason why plants generally look green. However, along with these two important pigments, there exist some other accessory pigments, such carotenes, that can be involved to a lower extent in the process of photosynthesis. The action spectrum of carotenes shows a blue absorption peak many times larger than the red peak, leading to an orange tint. Depending on the plant species, the relative importance of each pigment differs and the final action spectrum can therefore be seen as a combination of each individual action spectrum of the various pigments. We decided to use a global action spectrum, which will fit an averaged vegetation type. Such an action spectrum was adopted by the German Institute for Standardization and numbered DIN 5031-10 [39]. This action spectrum is shown by the gray line in Figure 2.

#### Scotopic spectral sensitivity

Scotopic vision occurs when luminance is below 10^−3^ cd/m^2^ [40]. This is clearly the case for stargazing conditions, and we decided to use scotopic spectral sensitivity as a basis for estimating star visibility. We retained the scotopic vision curve adopted by the commission international de l’éclairage (CIE) in 1951 [41]. This curve is shown in Figure 2: one important feature to notice on this curve is that the sensitivity peak is displaced towards the blue compared to the photopic curve. Basically, this means that for the same lumen, a lamp with a greater contribution in the blue part of the spectrum should generate more disturbance to stargazing and nocturnal vision of humans in general.

### Constant Lumen Normalization

In this work, one key element of spectral data processing was the normalization of all SPDs to a constant lumen output. We chose this normalization because most lamps are intended to illuminate the environment in order to favor human activity. By normalizing the entire lamp SPD with the photopic spectral sensitivity, all the resultant SPDs produce the same stimuli to the human eye (ignoring color variations from one lamp to another). We assumed that lighting is designed in such a way that the desired luminance (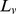) reaching the eye is higher or equal to the minimum luminance required for the photopic vision regime of the human eye. The photopic vision luminance threshold can be set to 0.6 cd.m^−2^ [42]. In fact, when using a unified system of photometry [43] based on a combination of photopic and scotopic luminances, we obtained pure photopic luminance when 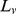 = 0.6 cd.m^−2^ or higher. Assuming a ground lambertian surface with a constant reflectance ρ, we were able to calculate the equivalent illuminance in lux (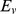) according to Equation 2:
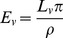
(2)


Assuming that luminance is higher or equal to 0.6 cd.m^−2^ and that for a typical summer city ground reflectance was 0.08 (based on asphalt reflectance from the NASA’s ASTER spectral library), at least 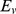
≈23 lux is required to be within the photopic vision regime. For a snow-covered surface this minimal illuminance is 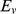
≈2 lux (snow reflectance of 0.98). This range of illuminance is representative of what is found on typical roadways.

The normalization method given by Equation 3 below defines the constant lumen SPD (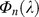)
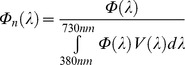
(3)where 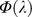 is the unnormalized SPD of the lamp and 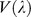 is the photopic spectral sensitivity function [44]. This function was derived from corrections to a previous revision of CIE 1931 - 2 deg color matching functions [45]. 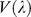 is shown on Figure 2. To calculate the constant lumen normalization, the integration spectral range (380–730 nm) was chosen to include significant spectral sensitivities of the biological processes covered by the present work. More specifically, we used the limits given by the PAS.

### Impact of Atmospheric Optics

Light sources usually have complex spectra and emit radiation in all directions. A proportion of the electromagnetic energy that is propagated upwards undergoes scattering and absorption processes before it is detected at the ground as diffuse radiation. It should be noted that the optical behavior of the diffuse radiation depends strongly on both the original spectra of the light source and its radiant intensity distribution as a function of the zenith angle 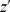 and azimuth angle 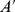. If 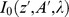 is the spectral radiance of an elementary surface, than the amount of electromagnetic energy delivered to and removed by an elementary atmospheric volume 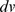 is
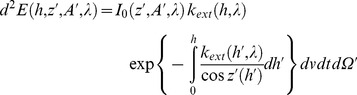
(4)where 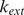 is the extinction coefficient of an atmospheric environment, 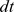 is a time interval, 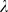 is the wavelength, and 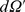
denotes an elementary solid angle in which the radiance of the ground-based light source is received at altitude 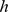. In Equation (4), we assumed that 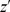 depends on the altitude due to vertical stratification of the air refractive index. For zenith angles smaller than 80 degrees and/or for short optical paths such a dependency can be neglected, and thus the factor 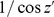 can be placed before the integral (in curly brackets). In all other cases a corresponding optical air mass should be used instead of 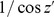. The exponential function in Eq. (4) is also called the transmission function:
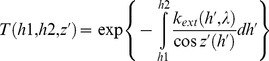
(5)and typically characterizes the attenuation of electromagnetic radiation along the beam path. It is evident that any increased atmospheric turbidity implies a more rapid intensity decay in the atmospheric environment. However, elevated turbidity conditions are closely related to the increased number of scattering domains, which also make the scattering processes more efficient. Since the elementary volume 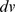 can collect light from all directions, the amount of energy scattered within the elementary solid angle 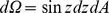 will be:
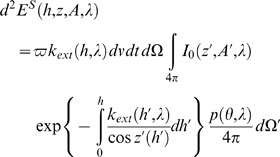
(6)where 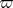 is the so-called atmospheric single scattering albedo [46], 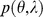 is the spectral scattering phase function, and 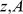 are the observational zenith and azimuth angles, respectively. The radiative energy scattered toward a hypothetical observer will be then given as:
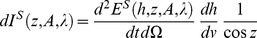
(7)since the photometry law dictates that the elementary amount of electromagnetic energy crossing an elementary surface 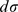 is by definition 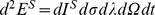. The scattered beam we considered in Equation (6) crosses the elementary volume 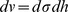 under the inclination angle 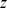, so the projection area is 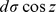 instead of 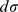 (consult Eq. 7). The total amount of scattered radiation received at a measuring point is obtained as an integral product of the spectral transmission function and elementary radiance, so:
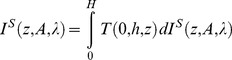
(8)where 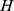 approaches infinity in a cloudless atmosphere. Under overcast conditions, 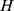 appears to be the altitude of the cloud base. The contribution of clouds to the spectral radiance is computed in the form of an additional term [47] (not shown here). One of most important parameters affecting the spectral radiance under overcast conditions is the spectral reflectance of the cloud. Green et al. [48] have shown that the average reflectance of a cloud is about 0.46, and we have used the same value in our computations.

In principle, 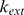, introduced in Equation (4), is a sum over all the extinction coefficients of the atmospheric constituents. However, we considered only aerosol and molecular extinction coefficients since these are dominant in a cloudless or undercloud atmosphere. Water vapor absorption was not considered because it becomes important only for wavelengths larger that 755 nm; in this work, we do not use wavelengths larger than 730 nm.

The optical thickness 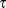 is used in the radiative transfer computations, rather than the extinction coefficient 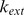. The relationship between 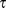 and 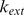 can be written as follows:
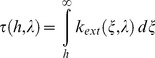
(9)


Here, 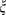 is an integration variable that characterizes the altitude about ground level. It is commonly accepted that Rayleigh’s optical thickness for the molecular atmosphere decreases exponentially with increasing altitude, 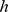, i.e.
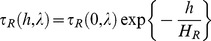
(10)where 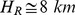 is the so-called scale height of the molecular atmosphere: it is the altitude up to which a homogeneous molecular atmosphere would extend. For the molecular optical thickness at ground level we used the following formula [49]:
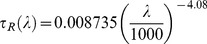
(11)with the wavelength measured in nm. The concept of scale height is also frequently applied for aerosols. However, the aerosol scale height 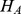 is much lower than that for the molecular atmosphere. For aerosols we can write:
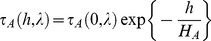
(12)where we employed 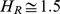
km. This value correlates well with the results obtained e.g. by Horvath et al. [50]. For a typical clear atmosphere, we assumed values of 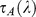 = 0. 2 at 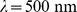 with an Ångström coefficient of 1.0, because a set of experiments conducted in urban and suburban regions have shown that the Ångström exponent is close to unity (e.g. [51], [52]). The total optical thickness is computed as 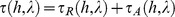.

The scattering angle 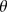 used in Equation (6) is determined from spherical geometry in the form:
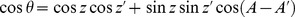
(13)


Once the scattering angle has been determined in this way, the scattering pattern for a turbid molecular-aerosol atmosphere can be expressed as follows:
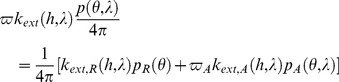
(14)


The expression given at the right hand side of Equation (14) has to be used in Equation (6). The molecular scattering phase function introduced with Equation (14) reads:
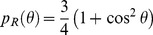
(15)while the aerosol scattering phase function is determined based on Mie theory:



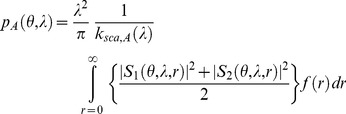
(16)In this equation, 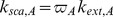, with 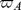 being the aerosol single scattering albedo. The dimensionless Mie functions 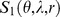 and 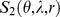 are weighted by a particle size distribution, 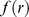, where 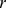 is the particle radius. It is expected that both 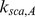 and 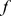
are altitude-dependent. The computations based on Equation (16) could be CPU-intensive if used in vast modeling. Therefore, some convenient approximations, such as the Henyey–Greenstein function [53],
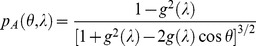
(17)are routinely applied in the use of radiative transfer tools. Although this function has no theoretical foundation, it can mimic the experimental scattering patterns quite well. The most significant advantage of this function is its analytical formulation. The asymmetry parameter 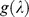 used in Equation (17) is the cosine-weighted integral of the scattering phase function. In conjunction with the single-scattering albedo 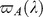, the asymmetry parameter 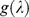 is the most important variable that is considered in any aerosol-related radiative problem. In principle, 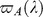 can vary from 0 to 1, while the acceptable theoretical values of 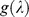 range from −1 to +1. Nevertheless, it has been well documented experimentally that typical values of 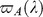 and 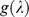 range from 0.8 to 0.9 in urban and suburban regions [50], [54]. We have used 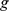 = 0.8 and 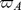 = 0.9 for the visible spectrum.

We considered that photon removal from the light fixture to the observer can be calculated by assuming that direct light does not reach the observer because of obstacles such as trees, buildings or topography, and that the main source of illumination at remote sites is derived from the light dome. This assumption can of course be weak for an astronomical site located on top of a mountain from where a light fixture can be seen in a direct line of sight.

### Reference Spectrum

In order to exclude any other parameter but wavelength, we decided to perform all calculations in reference to a standard SPD (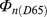). We chose the CIE D65 standard illuminant for this purpose. This spectrum, or daylight illuminant, corresponds roughly to a midday sun in Western/Northern Europe. We also applied the constant lumen normalization to that spectrum, as explained by Equation 3. We considered that biological evolution occurred under sunlight, and assumed that the CIE D65 illuminant is closely related to the three biological processes studied here.

### Spectral Indices Definition and Interpretation

Some attempt has been made in the past to define spectral indices related to photosynthesis and circadian action in the field of computer image rendering [55] and light and health research [56]. A melatonin suppression effect relative to HPS, and a ratio of the radiant power in a scotopic protected interval (440 nm - 540 nm) to photopic luminous flux, were also suggested in 2011 [30]. Starting from these anterior works, we set out to improve and standardize this concept, and to include the estimation of star visibility (scotopic vision). We also set out to explore the effect of the scattering of light through the atmosphere. In general, scattering processes may cause different effects in humans and other photosensitive species due to their different visual perceptions. Some species are even sensitive to the UV spectrum, as has been suggested in a recent study [57]. In this paper, the authors make a set of computations in order to estimate the real perception of three species by considering the spectral radiance produced by five different lamps. The findings are related specifically to the skyglow as perceived by humans (under scotopic vision) and two species of insects (one diurnal and one nocturnal). We suggested here three spectral indices ranging from 0 to ∼1 to characterize independently the potential spectral impact of a lamp SPD on the three following biological processes: 1- melatonin suppression, 2- photosynthesis, and 3- scotopic vision. To isolate the spectral properties from other variables, we first calculated a weighted lamp constant lumen SPD, where the weight function is the spectral sensitivity of the biological process considered. We then performed the same calculation for a CIE D65 constant lumen SPD in place of the lamp SPD. Finally, we computed the ratio of the weighted constant lumen lamp SPD over the weighted constant lumen CIE D65 SPD. When atmospheric effects were considered, the atmospheric transfer function was applied to the SPD of both constant lumens so that we could always compare the lamp with the CIE D65 illuminant under the same conditions. The integration limits were set to the spectral extent of the photosynthesis action spectrum (380 nm to 730 nm).

According to this, we can write the Melatonin Suppression Index (MSI), the Induced Photosynthesis Index (IPI) and the Star Light Index (SLI) in Equations 18, 19 and 20, respectively.
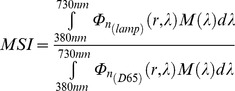
(18)

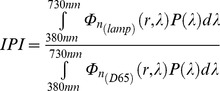
(19)

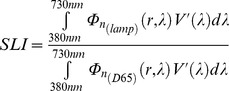
(20)


These indices can be easily calculated with the help of an OpenOffice spreadsheet file available at http://galileo.graphycs.cegepsherbrooke.qc.ca/lpds/index.php?n=Site.Products, if you have a SPD file to hand.

The three indices defined above enable the impact of the shape of the SPD to be evaluated. If we want to evaluate the global impact of a lamp we must also consider its illuminance. Since our indices use SPD normalized to a constant lumen (Eq. 3), we can thus obtain the global impact of a lamp by multiplying the indices by the illuminance of that lamp.

## Results and Discussion

### Spectral Indices for Direct Lighting

We first want to apply the above indices definitions to the special case of a direct sight towards a lighting device. By direct, we mean that we do not calculate any atmospheric effects. That special case does not only occur for direct sight, but can also be invoked when we observe a neutral reflecting surface lit by the lighting device. For example, when light trespass occurs, the light can enter a human eye after entering a bedroom by its window and then reflecting from a white wall. Table 2 shows the indices calculation results for a set of 13 lamps and for the CIE D65 illuminant. In our experiment, LED filtering was performed with the *Equilib* optical filter commercialized by Ledtech International. Together with the indices, we have provided in Table 2 the CCT approximation [58], CRI [59], x and y chromaticity coordinates [60], and typical spectral sensitivities (lumen/W). CCT, CRI, x and y were calculated for each lamp on the basis of their measured spectrum. The x and y chromaticity coordinates [60] for each lamp are given in Figure 1.

**Table 2:** Photometric characteristics of lamps under direct lighting.

	x	y	CCT	CRI	MSI	IPI	SLI	Typ. Lumen/W
LPS	0.58	0.42	1720	−47	0.017	0.380	0.088	100–200
HPS	0.53	0.42	2010	19	0.118	0.509	0.231	85
Metal Halide	0.36	0.38	4500	48	0.624	0.640	0.577	120
Halogen	0.43	0.42	3200	92	0.377	0.829	0.597	24
Incandescent	0.48	0.43	2600	93	0.255	0.923	0.490	15
Fluorescent T8 cool-white	0.40	0.39	3730	82	0.435	0.606	0.608	90
LED 5000 K	0.36	0.36	4440	61	0.542	0.636	0.617	100
Philips LED 4000 K	0.38	0.39	4100	63	0.452	0.623	0.563	75
LED 2700 K	0.44	0.39	2760	37	0.285	0.541	0.359	69
Nichia Amber	0.57	0.42	1720	47	0.043	0.682	0.170	53
Lumiled PC Amber	0.57	0.42	1720	36	0.046	0.610	0.154	59
LED 5000 K Filtered	0.47	0.46	2910	55	0.172	0.470	0.380	82
LED 2700 K Filtered	0.52	0.45	2260	34	0.077	0.443	0.230	61
CIE D65	0.31	0.33	6504	100	1.000	1.000	1.000	–

x and y are the chromaticity coordinates [52], CCT is the Correlated Color Temperature, CRI is the Color Rendering Index, MSI is the Melatonin Suppression Index, IPI is the Induced Photosynthesis Index and SLI is the Star Light Index.

If we consider HPS as a reference device with MSI = 0.12, we can note that only LPS (0.02), Nichia amber (0.04), Lumiled Pc Amber (0.05) and LED 2700 K filtered (0.08) have a reduced potential impact on melatonin suppression (their MSI values are below the HPS value of 0.12, as shown in Table 2). So, these lamps should be favored for nighttime lighting in order to restrict the potential impact of ALAN on human health. It is important to note that, during the night, metal halides have an impact more than five times higher than HPS (0.64/0.12 = 5.33). This means that if the two lamps have the same lumen output, the metal halide will be 5.33x more efficient in suppressing melatonin, assuming that for both lamps the luminance is high enough to suppress melatonin. Similarly we can show that LED 5000 K, Philips LED 4000 K and fluorescent lamps are respectively 4.5x, 3.75x and 3.6x more efficient at suppressing melatonin than is HPS. If we need lighting during the daytime, LPS, phosphor-converted amber LEDs and LED 2700 K filtered lamps should not be used; during the daytime, we need to select the highest MSI values. In our lamp selection, the maximum MSI was obtained using the metal halide lamp (0.62), followed by the LED 5000 K (0.54), Philips LED 4000 K (0.45) and fluorescent (0.43) lamps. None of these are as efficient as the Sun itself, here represented by CIE D65 (1.0).

Making the same analysis for the impact on photosynthesis using IPI values, we determined that only LPS, LED 5000 K-filtered and LED 2700 K-filtered lamps had a lower impact than HPS. The least beneficial lamp during the night is therefore clearly the incandescent lamp, with an index of 0.92, followed by the halogen lamp (0.83). However, during the daytime these lamps can be advantageously used for plant growth to compensate for shorter days and lower Sun illuminance in winter at high latitudes. This can also be helpful for greenhouse-based industries. Incandescent and halogen lamps showed a high IPI, but their lumen per watt ratio is low, so that even if they are efficient for photosynthesis per unit of lumen, they are not efficient for photosynthesis in terms of energy consumption. The IPI of phosphor-converted amber LEDs, metal halide and fluorescent lamps were found to be very similar (ranging from 0.61 to 0.68), even with very different SPDs (orange vs. white light). More specifically, phosphor-converted amber LEDs do not have any significant blue emission, in clear contrast to metal halide and fluorescent lamps (Fig. 3). However, if we look to the shape of PAS in Figure 2, we can see that both lamps have a similar IPI because phosphor-converted amber LEDs show a high emission in the region of the PAS red peak (∼660 nm). For metal halide, the SPD shows an important line that coincides well with the blue PAS peak (∼430 nm). Fluorescent light shows important lines in both the blue and red region.

The SLI is applicable to low illuminance conditions, which are basically satisfied during the nighttime. In this case, we used the scotopic spectral sensitivity to calculate the index so that it was not only applicable to the astronomical observation but more generally to low-light human vision. If again we take HPS as a reference (0.23), we can show that only LPS (0.09), phosphor-converted amber LEDs (0.17 and 0.15) and LED 2700 K filtered lamps (0.23) are better or equivalent to HPS for restricting the impact on observation of the night sky. LPS and phosphor-converted amber LEDs are better than HPS. The lamps with the most negative impact on night sky observation are, in decreasing impact order, LED 5000 K (0.62), fluorescent (0.61), halogen (0.6), metal halide (0.58) and Philips LED 4000 K (0.56). These lamps produce ∼2.5x more astronomical light pollution than HPS for the same lumen output. It is of particular concern that white LEDs show such a high spectral impact on the dark sky. Groups and communities involved in dark sky protection initiatives should be aware of that, given the attractiveness of this technology to lighting professionals and local authorities. LED is a long lasting technology (50,000–100,000 hours), and is relatively efficient and easily controllable/dimmable. In order to constrain astronomical light pollution or to increase star visibility while changing HPS to cool white LED lighting, we recommend reducing newly installed LED lumens by a factor of 2.5x compared to the HPS lumen originally installed.

### Spectral Indices for Indirect Lighting

**Figure 4:**
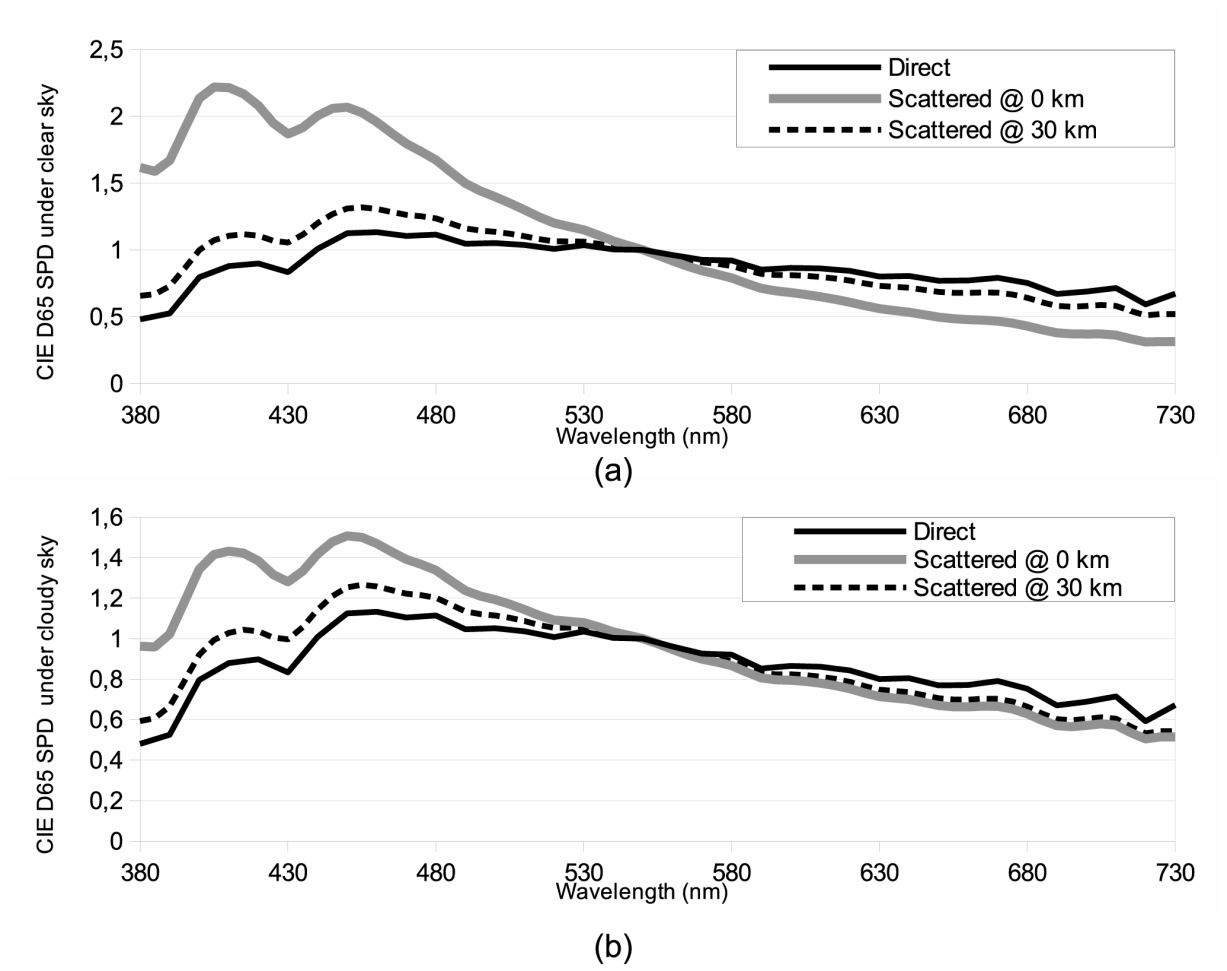
Spectral impact of the atmospheric transfer function as a function of the distance for the CIE D65 illuminant without cloud cover in (a) and with cloud cover in (b). Direct SPD relates to the original SPD before any atmospheric transformation. Compared to direct SPD, scattered SPD is bluer at short distances and redder at long distances.

We applied the atmospheric radiative transfer model described in the section ‘Impact of Atmospheric Optics’ to each lamp described in Table 2, and for a variety of distances from lamp to observer, ranging between 0 km and 30 km. This time, we did not observe the lamp directly but instead observed the light scattered by the surrounding atmosphere. It is important here to understand that the same model was applied to all SPDs, even the CIE D65. In other words, we compared the scattered light from a lamp with the reference CIE D65, both scattered to the same distance. In Table 3, we show the results for each index and for two distances (0 and 30 km). Atmospheric scattering increases the blue part of the SPD at 0 km. However, with increasing distance, the blue light is preferentially removed by extinction. This effect has a theoretical foundation and can be explained in terms of radiative transfer principles. If the distance from a hypothetical observer to the light source is small enough, the emitted light beams propagate through the atmosphere along short optical paths before they are backscattered toward the ground and detected by an optical device. Short optical paths imply a low attenuation due to extinction, and thus the scattering efficiency is by far the greatest modulator of downwelling radiation at surface level. Since an Ångström coefficient of 1.0 has been used in our numerical simulations, the aerosol optical thickness 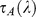 appears to be an inverse function of the wavelength. It is well-known that the Rayleigh optical thickness behaves like 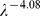 (Eq. 11), so molecular scattering definitely dominates in the blue part of the spectrum. In our model, the aerosol optical thickness is considered to be 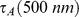 = 0.2 at the ground. Under these conditions, the Rayleigh optical thickness exceeds that for aerosols by 50% at 400 nm, but 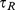 is almost three times smaller than 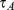 at 650 nm. In addition, the aerosol backscatter is much weaker than Rayleigh theory dictates (compare e.g. Plate 10.7 b in [61] with Fig. 2.3 in [62]). Both factors, the low extinction at short optical paths and the fairly efficient molecular backscatter, result in a specific spectral behavior of diffuse radiation in which Rayleigh and aerosol optics appear to be important. This coincides very well with our numerical computations, which show that diffuse irradiance behaves like 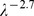 in the blue part of the visible spectrum. Thanks to a fairly steep decrease of 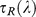 with increasing wavelength, the spectral behavior of diffuse irradiance shows a functional dependency 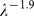 at the red edge of the visible spectrum. As the distance between an observer and light source increases, the situation changes significantly. The beams that form the light field near the measuring point are propagated at low elevation angles, thus traveling along long optical paths. In such a case, the aerosol optics become dominant due to the enhanced forward scatter and increased scattering efficiency. It should be noted that the ratio of forward to side scatter can exceed the value of 100 (or even more) for a polydispersed system of aerosol particles, while it is only 2∶1 in the case of Rayleigh scattering. For these reasons, Rayleigh scatter is considered to have a marginal effect on sky glow if observations are made at larger distances from the light source. The spectral dependency of diffuse irradiance therefore mimics a function of 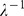 rather than 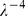, because the large optical path means that the light beams interact with an elevated number of aerosol particles. On the one hand, the higher number of scattering domains translates to an increased scattering efficiency, while on the other hand it results in a more efficient intensity decay, due to the exponential form of the transmission function (Eq. 5). One could say these two effects act against each other. In a very simplified case, the energy of the scattered radiation or radiance (Eqs. 6 or 8) could behave like 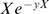, where 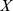 monotonically increases with growth in the optical path or number of scattering events (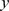 is an arbitrary scaling parameter). This function peaks at 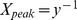, so the extinction exceeds the scattering efficiency if 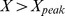, i.e. if the optical path is considerably large. The spectral dependency of diffuse irradiance is then further distorted, resulting in a functional behavior 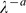 with 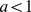. The numerical experiments performed in this paper have shown that 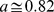 for distances of about d = 15 km, 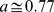 at d = 30 km, and 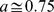 at d = 60 km. For very distant light sources, the coefficient 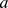 approaches its asymptotic behavior. The limit value of 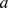 depends on the mutual interaction of the aerosol phase function and the extinction and scattering efficiencies. Figure 4 illustrates the complexities of the atmospheric effects. This figure shows the SPD of illuminant CIE D65 as a function of distance for two different atmosphere ((a) clear sky and (b) cloudy sky). All SPDs have been normalized at 550 nm to facilitate the comparison.

**Table 3:** Photometric characteristics of lamps after scattering into the atmosphere.

	Clear sky						Cloudy sky					
	MSI		IPI		SLI		MSI		IPI		SLI	
Distance (km)	0	30	0	30	0	30	0	30	0	30	0	30
LPS	0.008	0.014	0.241	0.349	0.053	0.078	0.012	0.015	0.317	0.359	0.072	0.081
HPS	0.099	0.113	0.342	0.465	0.188	0.218	0.107	0.114	0.430	0.477	0.210	0.221
Metal Halide	0.663	0.634	0.678	0.651	0.547	0.567	0.647	0.631	0.658	0.648	0.563	0.570
Halogen	0.329	0.364	0.592	0.761	0.544	0.583	0.350	0.369	0.718	0.779	0.571	0.587
Incandescent	0.203	0.241	0.572	0.820	0.431	0.474	0.225	0.245	0.759	0.846	0.461	0.478
Fluorescent T8 cool-white	0.403	0.427	0.549	0.594	0.561	0.595	0.417	0.430	0.579	0.598	0.585	0.598
LED 5000 K	0.500	0.533	0.601	0.631	0.598	0.610	0.517	0.536	0.618	0.633	0.608	0.611
Philips LED 4000 K	0.422	0.445	0.555	0.607	0.521	0.550	0.435	0.448	0.591	0.611	0.543	0.553
LED 2700 K	0.261	0.280	0.439	0.516	0.330	0.349	0.271	0.282	0.492	0.524	0.346	0.352
Nichia Amber	0.026	0.038	0.384	0.598	0.12	0.156	0.034	0.040	0.543	0.620	0.146	0.159
Lumiled PC Amber	0.034	0.042	0.357	0.540	0.107	0.140	0.039	0.043	0.491	0.558	0.131	0.144
LED 5000 K Filtered	0.136	0.162	0.336	0.437	0.315	0.362	0.151	0.165	0.406	0.446	0.349	0.367
LED 2700 K Filtered	0.056	0.071	0.288	0.403	0.173	0.214	0.065	0.073	0.370	0.415	0.203	0.218
CIE D65	1.000	1.000	1.000	1.000	1.000	1.000	1.000	1.000	1.000	1.000	1.000	1.000

For most of the lamps shown in Table 3, MSI, IPI and SLI are lower in scattered light compared to direct lighting indices. The only exception is the metal halide lamp for which MSI and IPI are slightly higher after scattering. This is probably because of the presence of a significant deep blue line at around 405 nm. No other spectra in our selection show such an important emission at a very low wavelength where scattering is very efficient. The same phenomenon is not observed for SLI, because the scotopic spectral sensitivity curve is almost zero at 405 nm.

**Figure 5:**
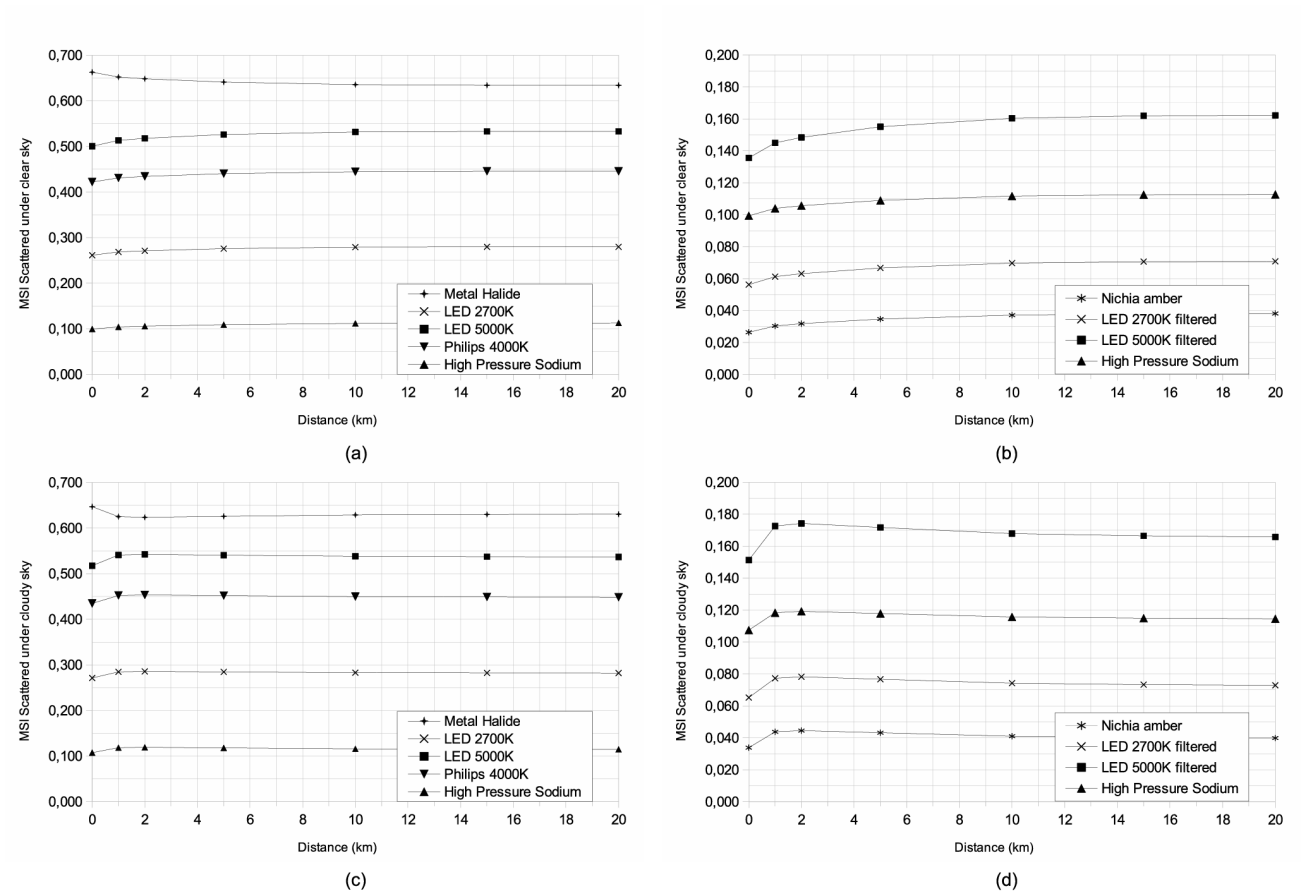
Atmospheric scattered Melatonin Suppression Index dependency, with distance between lamp and observer, (a) and (c) for usual street lamps and (b) and (d) for new technologies that reduce light pollution. The two upper plots (a) and (b) are for clear sky conditions, while (c) and (d) are for cloudy sky conditions.

**Figure 6:**
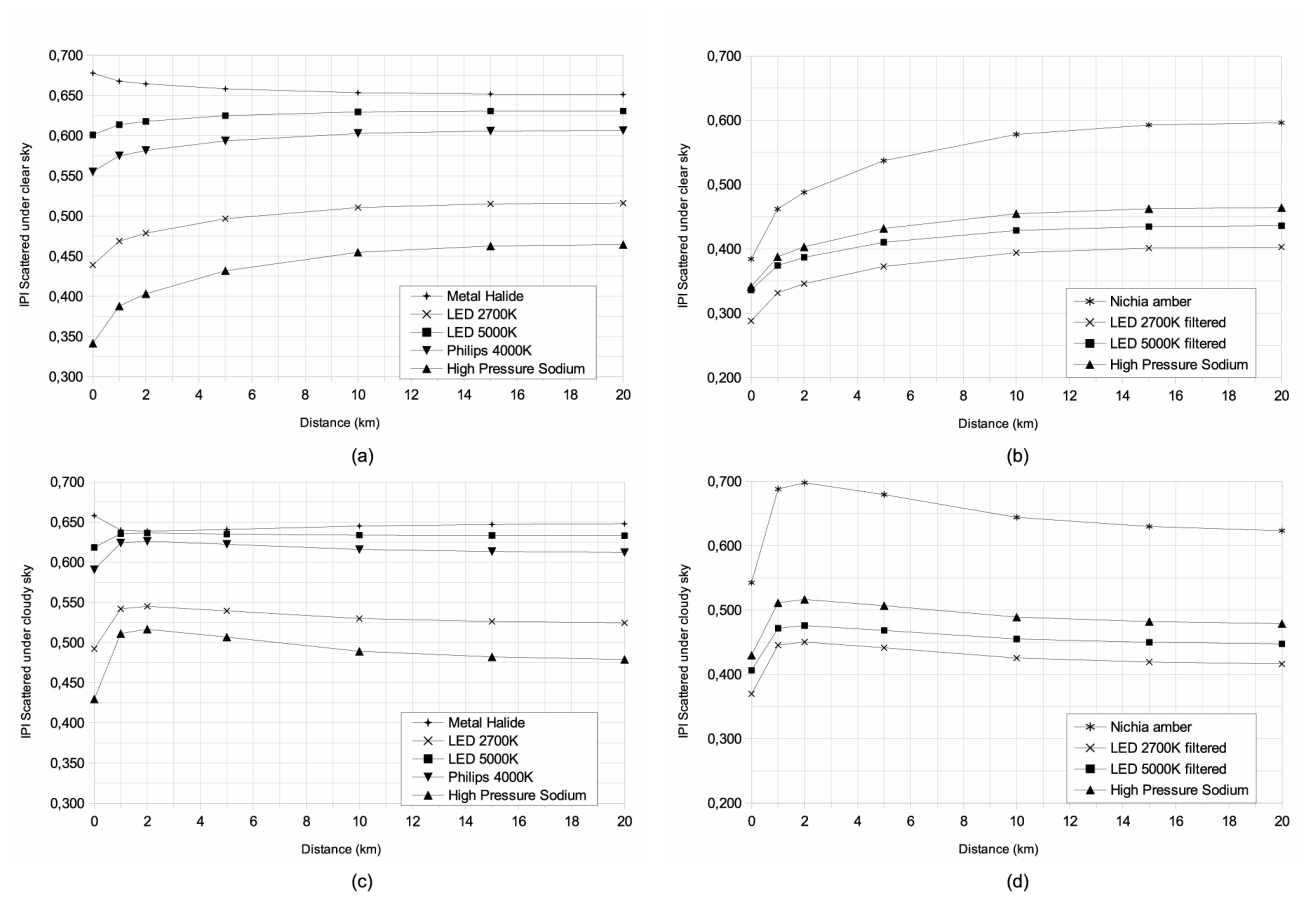
Same as Figure 5 but for Induced Photosynthesis Index.

**Figure 7:**
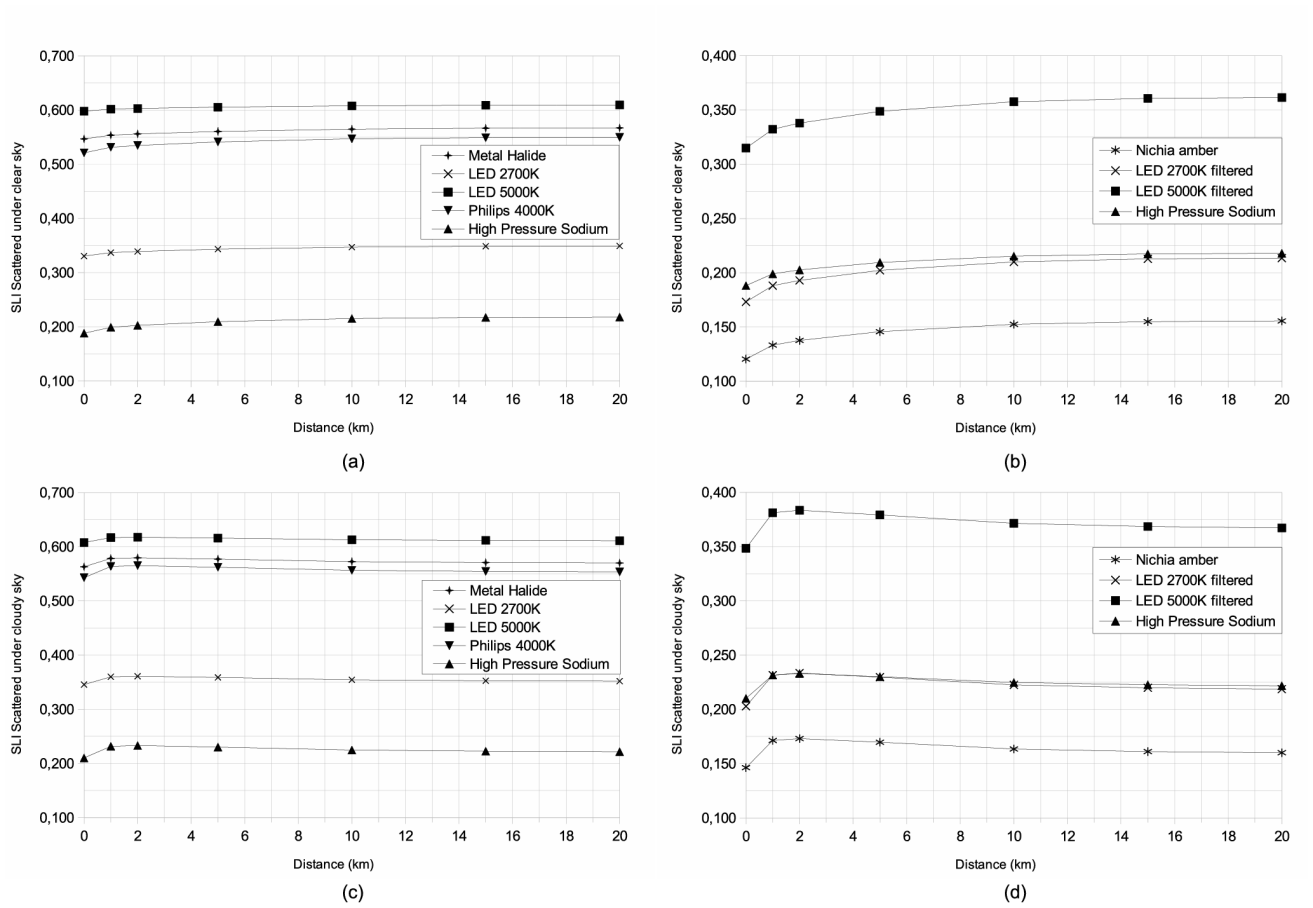
Same as Figure 5 but for Star Light Index.

To obtain a better understanding of the distance dependency of each index, we produced plots showing the indices as a function of distance (Figs. 5, 6, 7) under clear and cloudy sky conditions. These figures show the complex behavior of indices with distance. We separated each figure into two cases, namely new technologies intended to reduce light pollution in the right-hand plots (b and d), and the more standard technologies that are shown in the left-hand plots (a and c). For all plots, we showed the HPS case as a common reference. The two upper plots (a and b) are for clear sky conditions, while the cloudy conditions are shown in the lower two plots (c and d). Only lamps used in street lighting are plotted. For all cases the scattered indices become closer to direct values with increasing distance. Under cloudy sky conditions, all indices show a peak located at a distance of approximately 2 km. This kind of curve inflexion is not observed under clear sky conditions.

### Conclusions

In this paper, we introduced three new indices that can be used to characterize the SPD characteristics of any lighting device. These indices have been designed to allow a quick estimation of the potential impact of different lamp spectra on melatonin suppression, photosynthesis and star visibility. Indices have also been designed to separate the impact of the shape of the SPD from other factors, such as illuminance levels or the angular photometry of the lamp.

We used the indices to compare different lighting technologies in term of their spectral impacts. In particular we found that LPS, phosphor-converted amber LEDs and LED 2700 K-filtered lamps have a lower potential impact on melatonin suppression in comparison to HPS. LPS, LED 5000 K-filtered and LED 2700 K-filtered lamps show a lower impact on photosynthesis compared to HPS. Only LPS, phosphor-converted amber LEDs and LED 2700 K-filtered lamps are better than or equivalent to HPS in restricting the impact on star visibility. We also showed that atmospheric scattering under clear or cloudy skies generally reduces the value of the indices in comparison to direct light, except for metal halide lamps for which the effect is opposite.

During daytime, if we want to obtain the highest spectral impact, we should favor metal halide for increased melatonin suppression, while phosphor-converted amber LEDs and metal halide lighting is the best for stimulating photosynthesis.

It is important to realize that the spectral indices introduced here should not be considered alone in making a complete evaluation of the impact of a given lamp installation. As an example, a lamp with low MSI (low relative blue content) can impact significantly on melatonin suppression when the lamp illuminance is so high that the absolute blue flux becomes important. Basically, a global evaluation can be obtained by multiplying the index by the illuminance.

We hope that these indices will form some kind of standardization in characterization of lighting technologies, and that the lighting industry will provide such indices in the same way as they currently provide CCT or CRI, for example. Finally we consider that these indices will favor the design of new environmentally and health-friendly light devices, by providing a means of quickly evaluating the relative potential impact of a device at the design stage.
